# Remission of obsessive-compulsive disorder using ketogenic metabolic therapy in support of exposure and response prevention: a retrospective case report

**DOI:** 10.3389/fpsyt.2025.1555591

**Published:** 2025-06-20

**Authors:** Nicole Laurent, Katherine A. Tague

**Affiliations:** ^1^ Family Renewal, Inc., Vancouver, WA, United States; ^2^ Well Supported Behavioral Health, PLLC, Greenwich, CT, United States

**Keywords:** ketogenic metabolic therapy, ketogenic diet, obsessive-compulsive disorder, OCD, exposure and response prevention, ERP, metabolic psychiatry, case report

## Abstract

**Background:**

Obsessive-compulsive disorder (OCD) is a chronic and disabling condition that frequently resists standard interventions, including SSRIs and Exposure and Response Prevention (ERP). This case study explores the adjunctive use of ketogenic metabolic therapy (KMT) in conjunction with ERP for a 26-year-old man with treatment-resistant OCD characterized by the symmetry/ordering symptom dimension. The patient achieved remission of OCD symptoms and sustained improvements in mood, emotional regulation, and quality of life.

**Methods:**

A 26-year-old male with treatment-resistant OCD self-treated with a modified ketogenic diet and psychotherapy assisted ERP over 12 weeks. The diet featured a 1.5:1 macronutrient ratio (fat:protein+carbohydrates), self-monitored for nutritional ketosis (ketone levels ≥0.8 mmol/L), and included daily symptom tracking. ERP targeted symmetry/ordering-related compulsions. Symptom severity was assessed using the Dimensional Obsessive-Compulsive Scale (DOCS) and the Depression Anxiety Stress Scales (DASS-21). Long-term outcomes were evaluated over a 95-week follow-up.

**Results:**

Within three weeks of initiating KMT and subsequent ERP, daily compulsive behaviors decreased from 3–8 hours to less than one hour. Significant reductions in DOCS symmetry/ordering subscale scores were sustained at 95 weeks. Concurrent improvements were observed in emotional distress, measured by DASS-21, with all subscale scores normalizing by week 7. Qualitative feedback highlighted enhanced emotional regulation, sleep quality, and ERP engagement, attributed to the ketogenic diet.

**Conclusion:**

This case demonstrates rapid and sustained remission of OCD symptoms with the use of KMT and ERP. The findings suggest that KMT may provide a metabolic foundation that enhances the efficacy of ERP. Further research is warranted to explore the potential role of KMT in treatment-resistant OCD.

## 1 Introduction

Obsessive-compulsive disorder (OCD) is recognized by the World Health Organization as one of the top ten most disabling medical conditions worldwide (1). OCD is characterized by obsessions and compulsions; or recurrent intrusive, unwanted thoughts, images, or sensations accompanied by behaviors or rituals aimed at managing the distress caused by these intrusions (2). OCD has significant impacts on patients’ quality of life.

While the Diagnostic and Statistical Manual of Mental Disorders (DSM-5) does not separately categorize specific dimensions like of OCD (3) research has identified symptom dimensions within OCD, including symmetry/arranging, contamination/cleaning, and others—as clinically meaningful presentations (4, 5).

The symmetry dimension is highly prevalent, affecting approximately 86.8% of OCD patients in a large clinical sample (4). It is associated with an earlier onset of symptoms, more severe depressive symptoms, and the presence of sensory phenomena (4). These patients often do not report typical obsessions or fear specific consequences but are driven by a need to alleviate feelings of incompleteness or imperfection (4, 6).

The standard of care for OCD treatments includes cognitive behavioral therapies, specifically exposure and response prevention (ERP) and selective serotonin reuptake inhibitors (SSRIs) (7, 8). The data on ERP for OCD is promising, with one meta-analysis demonstrating statistically significant symptom remission through therapy alone, with approximately 65 percent of patients achieving remission. ERP showed large effect sizes compared to waitlist (1.31) and placebo (1.33), outperforming pharmacological interventions (effect size 0.55). ERP typically consisted of approximately 15 sessions delivered over about 13 weeks, though treatment protocols varied across studies (9).

SSRIs are the only validated monotherapy for OCD (10), but their efficacy is limited. Between 40% and 60% of patients fail to achieve full remission or clinical responses defined as a 25% or greater reduction in symptoms (7, 11). SSRIs are also associated with side effects, including apathy, hyperhidrosis, irritability, sexual dysfunction, and hot flushes (12). Clomipramine shows somewhat greater efficacy (13) but is linked to increased side effects, such as seizures, heart rhythm disturbances, dry mouth, constipation, urinary retention, and sexual dysfunction (14).

Given the limitations in both psychotherapeutic and pharmacological treatments, novel therapeutic strategies are needed for patients with treatment-resistant OCD. Exploration of the ketogenic diet as a nutritional metabolic intervention has raised questions about its potential relevance in addressing such cases (15).

In this case study, we examine the experience of a patient whose OCD was characterized by the symmetry/arranging dimension. This provides an opportunity to explore the potential of Ketogenic Metabolic Therapies (KMT) to target specific symptom dimensions and address gaps in current treatment approaches.

OCD has also been associated with elevated neuroinflammatory markers, such as cytokine IL-6 (16). In one study, OCD patients displayed as much as 20% to 30% higher neuroinflammation compared to healthy controls (17).

Inflammatory processes may contribute to the observed neurotransmitter imbalances characteristic of OCD. OCD is primarily associated with imbalances in glutamate and gamma-aminobutyric acid (GABA), disruptions in the GABA-glutamate pathway, elevated inflammatory markers, oxidative damage, and glucose hypometabolism. One study has shown lower glutamate levels in OCD patients, potentially indicating a neurochemical imbalance in excitatory neurotransmission (18). Abnormalities in neurotransmitter concentrations, such as glutamate and GABA, are thought to impair the cortico-striato-thalamo-cortical (CSTC) circuitry and MRI studies have revealed metabolic and structural abnormalities in this network (19, 20).

Such neurotransmitter dysregulation is often accompanied by increased oxidative stress. Similarly, OCD has been associated with elevated oxidative damage through imbalances in thiol/disulfide homeostasis, indicating elevated oxidative stress in individuals with OCD (21).

Oxidative stress may further exacerbate impaired energy metabolism observed in OCD. Neuroimaging studies indicate hyperactivity in the CSTC circuit in OCD, accompanied by reduced glucose metabolism in key brain regions. Hypometabolism has been observed in the caudate nucleus, a critical node in the CSTC loop, as well as in the insula and middle temporal gyrus (19). These metabolic abnormalities may underlie the cognitive inflexibility and executive dysfunction frequently reported in OCD patients (1).

Ketogenic diets have been discussed as a promising treatment for mental illness through modulation of multiple pathophysiological mechanisms (22) including neuroinflammation (23), neurotransmitter function (24), oxidative stress (25), and glucose metabolism (26). Conditions involving areas of brain hypometabolism and neuroinflammatory processes may benefit from the ketogenic diet’s ability to increase GABAergic transmission, reduce glutamatergic excitability, and decrease reactive oxygen species (ROS) production. Oxidative stress is mitigated by the diet’s antioxidant effects and enhanced mitochondrial function (27). By providing ketones as an alternative energy source, the ketogenic diet compensates for glucose hypometabolism in affected brain regions (28).

To our knowledge, this is the first published case study to report OCD symptom remission with a ketogenic diet and ERP treatment after unsuccessful symptom control with standard psychotherapy and medication.

## 2 Case presentation

### 2.1 Clinical background

The patient, a 26-year-old man, reported a psychiatric history marked by severe OCD symptoms. From approximately the age of10 to 21, he experienced distressing harm-related intrusive thoughts (e.g., themes of incest, pedophilia, and sexuality) and exhibited both obsessions and compulsions.

He had tried aripiprazole and propranolol during the course of his illness but discontinued due to adverse effects, including erectile dysfunction, night-time panic attacks, and an inability to drink alcohol socially without nausea and vomiting. Psychotherapeutic interventions, including psychodynamic therapy and CBT, were undertaken but did not result in significant symptom improvement.

After the age of 21, he believed his intrusive thoughts decreased significantly due to natural life exposures and OCD education, both of which were pursued independently without concurrent standard psychotherapy or medication. Natural Life Exposure refers specifically to increased engagement in everyday activities and responsibilities previously avoided due to OCD symptoms, such as attending classes, participating in social interactions, and managing daily routines. His compulsions, however, particularly those related to symmetry, order, and perfectionism, persisted. Physical compulsions included maintaining extreme order and symmetry in his living space and on his digital devices. He also engaged in complex verbal rituals, averaging 30 to 120 minutes a day, with occasional peaks of well over three hours during periods of heightened distress. These rituals were highly intricate and required precise execution, with any perceived errors necessitating repetition until performed “correctly.”

The patient self-initiated a ketogenic diet, and eight days later established treatment with an ERP therapist. At that time, the therapist confirmed a diagnosis of OCD with the qualifier of mixed obsessional thoughts and acts (ICD-10 code F42.2), indicating the presence of both intrusive, distressing thoughts (obsessions) and repetitive behaviors or mental acts (compulsions) performed to alleviate distress or prevent feared outcomes. Specifically, ERP began with psychoeducation, cognitive restructuring, and hierarchy development four weeks after KMT initiation, and formal exposure exercises started one week later at five weeks. Between the week 7 assessment and the nearly two-year follow-up (week 95), the patient independently maintained the ketogenic diet without further structured dietary supervision, monitoring, or additional psychotherapy sessions. ERP was not previously attempted, and no other forms of psychotherapy were provided concurrently. He adhered to both the diet and the ERP treatment consistently.

**Table 1** provides a detailed chronological overview of assessments, symptom severity scores, and intervention events throughout the treatment period.

**Table 1:** KMT-centered timeline of assessments and intervention events.

Time Since KMT Initiation	Event	DOCS Symmetry Score	DASS-21 Total	DASS-21 Subscales)	Clinical Interpretation
Day 0	Ketogenic Diet Initiated	–	–	–	Patient independently began KMT to address long-standing OCD symptoms. No additional interventions at this time.
8 Days Later	Initial Symptom Assessment Conducted	12	32 (Moderate Distress)	Depression 10 Anxiety 12 Stress 10	First clinical documentation of symptom severity post-KMT initiation. No structured psychological interventions initiated at this point.
1 Week Later	Nutritional Ketosis Confirmed	–	–	–	First documented ketone measurement: 2.3 mmol/L, confirming ketosis.
3 Weeks Later	Follow-Up Symptom Assessment (Significant KMT-Driven Improvement)	Reduced to 7	Reduced to 6 (Normal Range)	2/2/2	Patient reported marked symptom reduction attributed to KMT alone, stating OCD symptom burden and emotional distress significantly decreased before additional treatments were introduced.
4 Weeks Later	Formal Initiation of ERP (Pre-Exposure Phase Begins)	–	–	–	Psychoeducation, cognitive restructuring, and hierarchy development introduced as a secondary intervention to KMT. No exposure exercises were conducted at this stage.
5 Weeks Later	Behavioral Therapy Progression (ERP Exposure Introduced as Adjunct to KMT)	–	–	–	A therapist-led behavioral component was incorporated after metabolic therapy had already led to substantial improvement. No additional symptom assessments were performed at this stage.
7 Weeks Later	Follow-Up Symptom Assessment (KMT Sustaining Gains Post-ERP Exposure Introduction)	Reduced to 5	Reduced further to 4 (Normal Range)	1/1/2	Patient reported KMT continued as the foundation for symptom control. Symptom remission remained stable after additional behavioral therapy was introduced.
Nearly 2 Years Later	Long-Term Follow-Up Assessment	2 (Sustained Recovery)	2 (Sustained Recovery)	0/1/1	Sustained remission observed nearly two years post-intervention, with KMT initiated prior to ERP.

### 2.2 Ketogenic metabolic therapy intervention strategy

The patient had independently discovered information about ketogenic diets as a potential treatment for mental illness. After extensive research, he decided to implement it in the hope of alleviating his debilitating and chronic symptoms of OCD. He did not pursue medical oversight as he had no known comorbid medical conditions and was not taking any medications, nor did he seek professional guidance from a dietary professional. He did, however, inform his ERP psychotherapist that he was on a “low carb” diet, who then warned him that ketogenic diets had poor long-term outcomes for mental and physical health. As the patient was already experiencing treatment effects, they did not abandon the treatment based on this discussion.

Before starting KMT, he frequently ate fast food from national chains, typically three or four times a week, and highly processed sweets once or twice a week. Regular meals included homemade breakfast sandwiches on processed bread, peanut butter, and sugar-sweetened jelly, and pasta dishes with animal protein for dinner. His overall dietary pattern before KMT featured significant amounts of refined carbohydrates and added sugars, and relied heavily on highly processed foods.

Macronutrient ratios were initially set at a modified-ketogenic ratio of 1.5:1 (187 g fat, 150 g protein, 30 net carbohydrates). Average percentages from macronutrients were 71% fat, 24% protein and 4% carbohydrates. These macronutrient percentages were determined independently by the patient, who systematically tracked dietary intake daily using a personal spreadsheet. The diet included protein sources such as eggs, pork, chicken, and hot dogs; fats from coconut oil, full-fat dairy, avocado products, and peanut butter, and carbohydrates from low-carb vegetables, nuts, and small amounts of berries. He started with three ketogenic meals a day but naturally shifted to two meals, mid-morning and evening, creating a 16–17 hour daily fasting period. He also added a monthly 24-hour fast.

Self-initiated supplementation included omega-3 fatty acids (EPA/DHA ratio of 1,290 mg to 620 mg), electrolytes from Morton Lite Salt (providing sodium, potassium chloride, and potassium iodide), magnesium glycinate or threonate. N-acetylcysteine (NAC) with selenium was added exactly one month after starting KMT.

**Figure 1:**
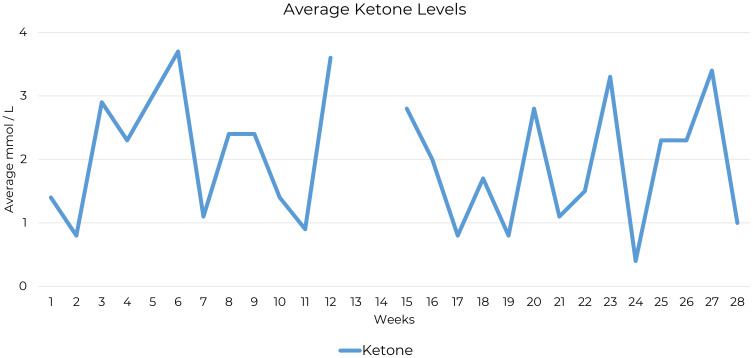
Average weekly ketone levels over 28 weeks. This graph shows weekly average ketone levels (mmol/L) over 28 weeks in a single OCD patient following a ketogenic diet.

Testing was carried out one to three times a week from Week 1 to Week 28, with 93% compliance. Daily ketone measurements during the 28-week testing period were made using the Keto-Mojo® GK+ Blood Glucose and β-Ketone Dual Monitoring System, which showed initial nutritional ketosis achieved at 2.3 mmol/L. Ketone measurements for weeks 13 and 14 were missing due to a temporary shortage of test strips. However, the patient reported consistently adhering to the ketogenic diet during this period (**Figure 1**) and through all periods of symptom assessment.

## 3 Evaluation of intervention outcomes

### 3.1 Quantitative outcomes

Depression and anxiety significantly contribute to compulsive behaviors in individuals with OCD, affecting their quality of life (29, 30). Addressing depression in clinical practice enhances patient safety and well-being. Assessing mood symptoms in OCD patients is recommended (31) to evaluate suicide risk and coexisting depressive symptoms, helping reduce morbidity and mortality (32). The patient’s psychotherapist therefore conducted standardized mood assessments alongside OCD symptom evaluations at the start of ERP treatment and at additional time points.

The Depression Anxiety Stress Scales (DASS-21) have been validated across diverse populations, demonstrating robust internal consistency and construct validity. It measures emotional symptoms of depression, anxiety, and stress, offering insight into patients’ emotional status and aiding clinicians in tracking mood symptoms over time (33). Severity classifications for the DASS-21 subscales are depression (normal 0 to 4, mild 5 to 6, moderate 7 to 10, severe 11 to 13, extremely severe 14 or greater), anxiety (normal 0 to 3, mild 4 to 5, moderate 6 to 7, severe 8 to 9, extremely severe 10 or greater), and stress (normal 0 to 7, mild 8 to 9, moderate 10 to 12, severe 13 to 16, extremely severe 17 or greater). The total score is not classified by severity, requiring interpretation of subscale scores to assess emotional distress (34).

At baseline, the patient’s DASS-21 assessment showed a total score of 32. While the total score provides a general indication of overall distress, severity classifications are determined based on individual subscale scores. The subscale scores were depression 10, anxiety 12, and stress 10, classified as mild, moderate, and normal, respectively. These subscale classifications reflect varying levels of distress in each domain, emphasizing the importance of interpreting the DASS-21 results based on individual subscales rather than the total score (35).

**Figure 2:**
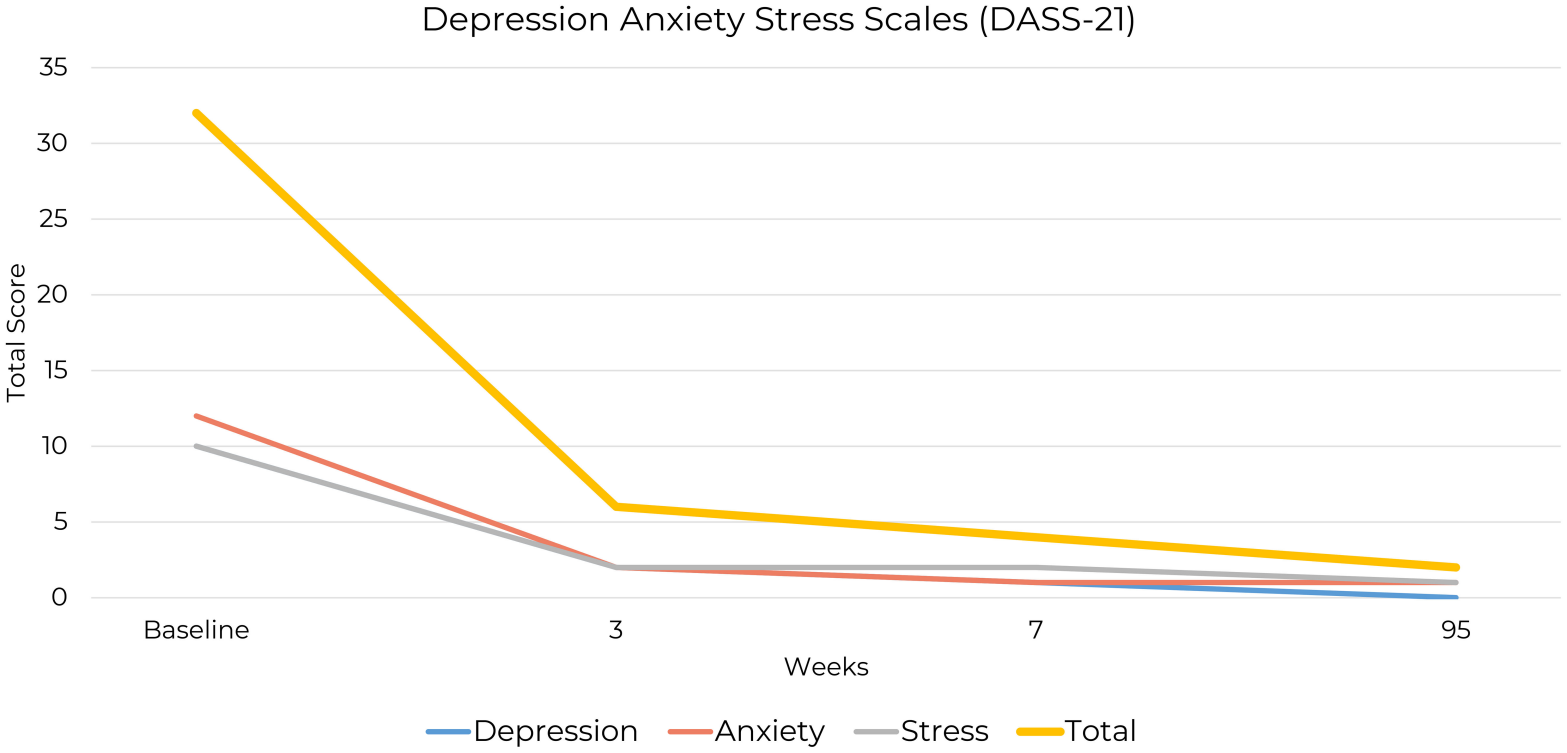
Changes in DASS-21 total and subscale scores from baseline to follow-up.

By Week 3, the patient’s total score had decreased to 6, with subscale scores of depression 2, anxiety 2, and stress 2, all within the ‘normal’ range for each subscale, reflecting minimal distress. By Week 7, his total score had further decreased to 4, with subscale scores of depression 1, anxiety 1, and stress 2 demonstrating continued improvement. The final score at extended follow-up was 2, which remained well within the ‘normal’ range, with subscale scores of depression 0, anxiety 1, and stress 1, indicating sustained recovery over a nearly two-year period (**Figure 2**).

The Dimensional Obsessive-Compulsive Scale (DOCS) is a self-report measure assessing OCD symptom severity across four dimensions: contamination/washing, harm obsessions/checking compulsions, symmetry/ordering, and unacceptable thoughts (36). The DOCS provides a dimensional approach to OCD severity, independent of obsession content. It uses time-based scales to measure daily symptom engagement and severity, ranging from “none at all” to “extreme,” allowing for nuanced quantification of OCD symptoms across dimensions of both temporality and intensity.

Psychometrically, the DOCS has demonstrated strong reliability and validity, showing sensitivity to treatment effects, with specific cutoff scores identified to differentiate between individuals with OCD, other anxiety disorders, and non-clinical groups. A cutoff score of 21 was found to correctly classify approximately 70% of OCD patients and 70% of patients with other anxiety disorders (36, 37). The summed score of the four subscales is reported to have the best diagnostic utility, suggesting that while the subscales assess distinct facets of OCD, their combined score is a more reliable indicator of total OCD severity (38).

It is of clinical note that the patient’s highest total DOCS score was 12, which falls below the cutoff score of 21 used to differentiate OCD from other conditions. However, this total score did not fully capture the severity of the patient’s symptoms, as they were exclusively confined to the symmetry/order dimension. The low total score is a result of minimal symptom expression in the other three dimensions assessed by the DOCS. Therefore, in this case, the subscale score provides a more accurate reflection of the patient’s condition at baseline (**Supplementary Table 1**).

**Figure 3:**
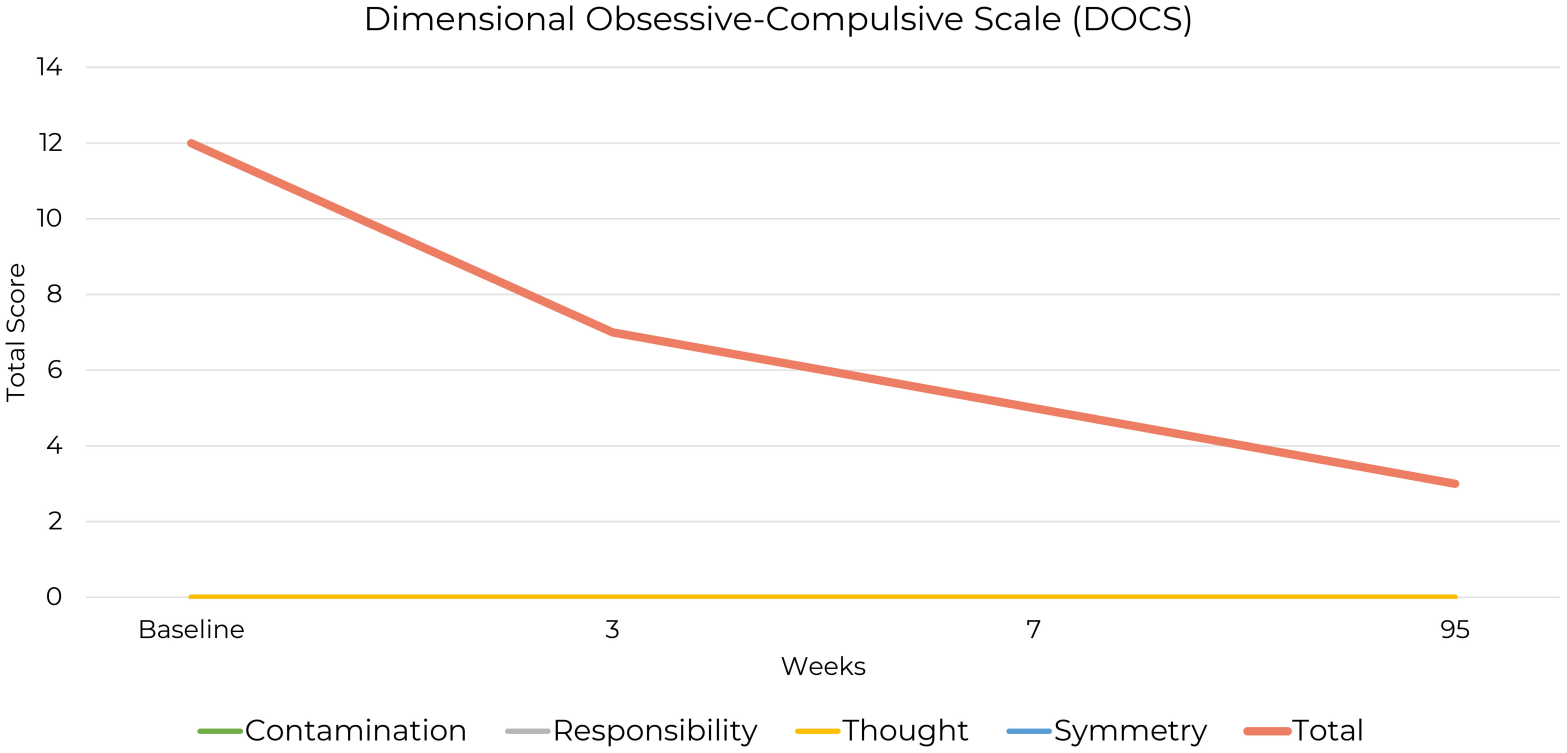
Changes in DOCS Symmetry Scale Scores from baseline to week 95.

The symmetry/order subscale of the DOCS provided detailed insights into the patient’s symptom severity at baseline, three-week, seven-week and 95-week follow-up assessments (**Figure 3**).

He scored 12 out of 20, indicating significant life disruption. At baseline, he reported spending between three and eight hours a day on symmetry-related thoughts and behaviors. By the first follow-up, this had decreased to under an hour per day, a reduction that was sustained up to the 95-week follow-up, reflecting a substantial and lasting decrease in the impact of these obsessions and compulsions.

Avoidance of symmetry-related situations remained minimal, with reports of “none at all” at baseline, briefly increasing to “little” at the first follow-up, then returning to “none at all” by the extended follow-up. Distress from the perception of things being “not just right” decreased from “severe” to “mild,” indicating partial but sustained anxiety reduction. Disruption to daily routines improved from “severe” to “none at all,” reflecting functional recovery. Difficulty dismissing symmetry-related thoughts and resisting urges decreased from “severe” to “little” at both follow-ups, showing improved control over compulsive behaviors.

### 3.2 Qualitative outcomes

Qualitative data revealed the patient’s view on the complementary roles of the ketogenic diet and ERP in recovery. He attributed improved emotional regulation, stabilized energy, and sleep quality to the ketogenic diet, distinct from ERP’s focus on obsessive-compulsive behaviors. He stated “ERP taught me how to break decades of ‘habit’ [order & symmetry]. It gave me ‘permission’ to stop. The ketogenic diet, however, was the foundation of this recovery. It empowered me. It is extremely challenging to integrate counseling when your brain is not working properly.” He added, “I state with absolute certainty that the ketogenic diet saved my life.”

## 4 Discussion and conclusion

This case presents the patient’s recovery process as the result of the combined interventions, of ERP and a ketogenic diet. ERP effectively targeted compulsive behaviors related to symmetry and order, while the patient also reported broader improvements such as stabilized energy levels, better sleep quality, reduced generalized anxiety, and a sustained sense of calm and presence. These outcomes were described by the patient as occurring in the context of using a ketogenic diet.

Although the ketogenic diet and ERP were initiated within the same general treatment period, the distinct timeline of their implementation and the sustained remission observed over extended follow-up suggest the possibility of complementary mechanisms at play. This case may introduce the potential for the ketogenic diet to provide a neurological and metabolic foundation that supports the goals of ERP, allowing patients to engage more effectively with therapeutic interventions. Future research should explore the ketogenic diet as an adjunctive treatment in ERP, investigating its role in augmenting outcomes and facilitating recovery in OCD and related conditions.

The patient was not taking OCD medication at the time of implementation, but he did report prior medications had “worsened” symptoms. Together, KMT and ERP appeared more effective in reducing symptoms than previous psychotherapy and psychotropic intervention.

This case study offers insights into KMT as a potential intervention for OCD but has some limitations. As a single-subject study, the findings lack generalizability. The design does not allow causal inferences, as symptom improvements could result from uncontrolled variables such as placebo effects, ERP, or psychological factors. The patient’s self-initiated use of NAC, a supplement commonly explored for OCD-spectrum conditions due to its antioxidant and glutamatergic modulation effects (39), adds complexity in interpreting the outcomes.

Although KMT and ERP were implemented within overlapping treatment periods, the five-week gap between the initiation of KMT and the commencement of ERP’s exposure component introduces a limitation, as it complicates efforts to distinguish the potential contributions of each intervention to the reported outcomes; however, it is notable that improvements in mood and OCD symptomatology were reported prior to the active implementation of exposure techniques. Self-reported data may introduce bias or variability, and the absence of a control group complicates isolating the effects of ketogenic therapy from natural symptom fluctuations or external influences.

Future research in this patient population should include qualitative data and assessment to accurately reflect the level of distress and impairment experienced. This need is reflected in this case study, in that the DOCS score alone suggested only subclinical levels of distress. Additional qualitative assessment revealed that the disorder significantly affected the patient’s life beyond the indication of the baseline DOCS score. Furthermore, due to the complex etiology of OCD, future studies should investigate whether these findings can be replicated in larger OCD populations and examine the effects of KMT without the added variable of ERP. This recommendation reflects the need to determine whether KMT alone produces clinically significant symptom improvements, clarifying its therapeutic value independent of psychotherapy.

According to diagnostic criteria, the patient’s post-intervention reduction in obsessive-compulsive symptoms to less than one hour per day represents a clinically significant improvement, potentially reducing the symptom severity below the diagnostic threshold (3).

This study contributes to the existing evidence base of metabolic psychiatry and the role of a ketogenic diet as a potential transdiagnostic treatment for mental illness (40). This case study provides a real-world example of ketogenic therapy as an adjunct to ERP for OCD recovery and introduces the concept that KMT may be a potential vehicle for creating the neurological and metabolic foundation necessary for the difficult therapeutic work of existing interventions, such as ERP.

## Data Availability

The original contributions presented in the study are included in the article/**Supplementary Material**. Further inquiries can be directed to the corresponding author.

## Supplementary material

Supplementary Table 1 Changes in Dimensional Obsessive-Compulsive Scale (DOCS) Subscale Items and Scores from Baseline to Extended Follow-Up. The patient’s symmetry/ordering symptoms demonstrated clinically meaningful improvement, with severity decreasing from baseline to the one-month reassessment and maintaining a reduced level at the 95-week follow-up.
