# Supplementary material for: Remission of obsessive-compulsive disorder using ketogenic metabolic therapy in support of exposure and response prevention: a retrospective case report
Source: Front Psychiatry. 2025 Jun 20;16:1555591. doi: 10.3389/fpsyt.2025.1555591 (PMC12231482; doi:10.3389/fpsyt.2025.1555591)
Supplement: Supplementary Table 1 — Changes in Dimensional Obsessive-Compulsive Scale (DOCS) Subscale Items and Scores from Baseline to Extended Follow-Up. The patient’s symmetry/ordering symptoms demonstrated clinically meaningful improvement, with severity decreasing from baseline to the one-month reassessment and maintaining a reduced level at the 95-week follow-up. [file Table1.docx]

## Supplementary Table 1: Changes in Symmetry/Ordering DOCS Subscale Scores from Baseline to Extended Follow-Up

| **Dimensional Obsessive-Compulsive Scale (DOCS) Item - Symmetry/Order Subscale Items** | **Baseline** | **Week 3** | **Week 7** | **Week 95** |
| --- | --- | --- | --- | --- |
| About how much time have you spent each day with unwanted thoughts about symmetry, order, or balance and with behaviors intended to achieve symmetry, order or balance? | Between 3 and 8 hours per day | Less than 1 hour each day | Less than 1 hour each day | Less than 1 hour each day |
| To what extent have you been avoiding situations, places or objects associated with feelings that something is not symmetrical or “just right?” | None at all | None at all | Little | None at all |
| When you have the feeling of something being “not just right,” how distressed or anxious did you become? | Severe | Little | Little | Little |
| To what extent has your daily work routine (work, school, self-care, social life) been disrupted by the feeling of things being “not just right,” and efforts to put things in order to make them feel right? | Severe | Extreme | Little | None at all |
| How difficult is it for you to disregard thoughts about the lack of symmetry and order, and refrain from urges to arrange things in order or repeat certain behaviors when you try to do so? | Severe | Extreme | Little | Little |

The patient's symmetry/ordering symptoms demonstrated clinically meaningful improvement, with severity decreasing from baseline to the one-month reassessment and maintaining a reduced level at the 95-week follow-up.
